# Bioecological and Behavioral Interaction between Pollinating Bees and the Pioneer Shrub *Ludwigia nervosa* in Degraded Area Suggests an Exotic Bee as Its Major Pollinator

**DOI:** 10.3390/biology10020114

**Published:** 2021-02-04

**Authors:** Eliana Aparecida Ferreira, Samuel Boff, Sandra S. Verza, Rosilda Mara Mussury

**Affiliations:** 1 Laboratory of Pollinators, Faculty of Biological and Environmental Sciences, Federal University of Grande Dourados, Dourados-Itahum Highway, 12th km, Dourados 79804-970, Mato Grosso do Sul, Brazil; lih.ferreira.ivi@gmail.com (E.A.F.); sandraverza@yahoo.com (S.S.V.); 2 Department of Animal Ecology and Tropical Biology, University of Würzburg, Am Hubland, 97074 Würzburg, Germany; samboff@gmail.com

**Keywords:** cross pollination, disturbed humid area, germination speed, honey bees and native bees, pollen limitation

## Abstract

**Simple Summary:**

*Ludwigia nervosa* plants are commonly found in humid environments in the process of regeneration; however, little is known about reproductive contribution to attracting bees in fragmented environments. Therefore, we aimed to identify the bees visiting the flowers, and whether during pollen and/or nectar collection they performed pollination. The absence of pollen limitation shows that there was sufficient and efficient deposition of pollen in *L. nervosa* flowers during the visit, guaranteeing the formation of larger fruits and seeds with high germinative potential.

**Abstract:**

The flowers of plants of the genus *Ludwigia* are an important source of food for several species of bees. In the current study, we conducted an experiment with the aim to describe the reproductive biology and phenology of *L. nervosa*; to identify the species of visiting bees; analyze the foraging behavior of bees; and to investigate whether the reproductive success of the species is related to the foraging activity of bees. We found that the flowers received visits from several native bee species (n = 7), in addition of the exotic honey bees which came to be the dominant species. During visits the majority of the bees foraged in both resources, pollen and nectar. The significantly higher production of fruits in open pollinated pollination experiment compared to artificial cross pollination, suggests honey bees as effective pollinator of this plant species in the study site. Pollen deposition occurs efficiently, given the absence of pollen limitation. Despite massive visitation of honey bees, *Ludwigia* *nervosa* is attractive to native bees, and therefore it may help to sustain population of both native and exotic pollinators in fragmented humid areas.

## 1. Introduction

In ecological regeneration, pioneer plants have key role, as they prepare the environment in the regeneration process for colonization by later successional species [1]. Among pioneer species in the Neotropical region, those belonging to the Onagraceae family (Myrtales) are often emphasized due to the abundance of species in the family, and because they are an important source of floral resources for several species of bees [2,3,4].

Regarding the Onagraceae species, 45% of the total species are allogamous [5]; however, different forms of cross-fertilization have been observed, such as hercogamy, male sterility, dichogamy, and self-incompatibility [6]. Self-incompatible plants are more vulnerable to the effects of fragmentation [7,8] since fruit production is influenced by the diversity of floral visitors, the quality of forages, the availability of resources, and human interference [9]. Floral visitors guarantee greater reproductive success, with the formation of more fruits and seeds [10]. Several genera of bees, such as *Diadasina*, *Ptilothrix*, *Tetraglossula*, *Pseudagapostemon* [11,12,13,14,15], *Xylocopa* [16], and *Bombus* [10], were shown to forage in flowers of *Ludwigia*, a diversified genus in Onagraceae.

The *Ludwigia* genus, with 82 described species, shows a Pantropical distribution [17]. The genus has the defining characteristics of pioneer plants, especially when it comes to surrounding rivers, streams, and wet or flooded environments [18]. *Ludwigia nervosa* (Poir.) H. Hara, is a perennial shrub with primary distribution in wet, open, and regenerating areas [2,13,19]. Additionally, because of its continuous flowering habit, the plant has potential to provide food (pollen and nectar) for a wide array of pollinators throughout the year [20].

The flowers of *Ludwigia* produce pollen grains in tetrads connected by viscin threads [21]. The bees specialized in collecting pollen resource from *Ludwigia* species have rigid, long, and branched bristles in the scopa [12]. Moreover, efficient pollination is associated with factors such as frequency of visits, pollen and nectar collection behavior, and body size [22].

Commonly *Ludwigia* species occurs in swampy area that is undergoing a natural regeneration process. The generalized floral shape of *Ludwigia* flowers is suggested to trigger flower visitation by different bee species stimulating the reproductive success of the species in poor environments. In order to understand the role of pollinators in flowers of *Ludwigia* in anthropized environment we performed observations of local floral visitors with the aim to characterize floral resources foraged by visitors and pinpoint the most important group of pollinators based on foraging behavior and frequency of visits. Moreover, we performed reproductive (pollination) experiments simulating high displacement of pollinators across flowers and assessed the reproductive success between treatments through comparison of fruit set, fruit length, germination speed, and comparing the growth rate of newly germinated individuals. Following the plant population through the entire season gave us also the possibility to describe its phenology.

## 2. Materials and Methods

### 2.1. Sampling Area and Plant Population

**Figure 1:**
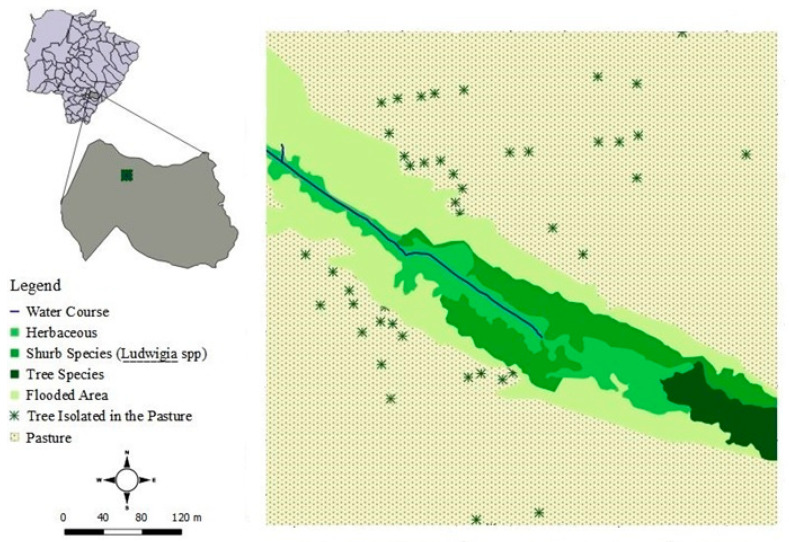
Map of the study site and distribution of *Ludwigia* spp.

The study was performed in 72 individuals on the banks of “Córrego Zézão,” in the city of Ivinhema, Mato Grosso do Sul (22°16′0.00″ S, 53°52′37.0″ W), Brazil, between October 2015 and September 2016. The climate in the region is subtropical, ranging from humid to sub humid [23]. The study site covered area of 26,500.00 m^2^, which is part of a fragment of riverside forest with a total area of 60,025.29 m^2^, which in the natural regeneration process. The area has a predominance of swamp with herbaceous vegetation (grasses and ferns) and areas in the early stages of regeneration (secondary succession) with shrubs plants (e.g., *Miconia* sp. (Melastomataceae) and *Ludwigia* spp.) sparsely distributed (Figure 1). Previously, this area, which corresponded to the stream’s permanent protection zone, was impacted by the fragmentation resulting from the anthropization of the region. Currently, with no direct human interference on the site, this area is in the process of regenerating natural vegetation.

The focal plant species was identified by a specialist in the laboratory of Applied Botany, and the specimens were deposited at the Herbarium of the Federal University of Grande Dourados—UFGD, with the following registration numbers: 6390—*L. nervosa*. The Brazilian National Research Council (CNPq)/Council of Genetic Heritage Management (CGEN/MMA), under the number A9ECAC6, authorized the collection of the botanical material.

### 2.2. Flowering and Fruiting Phenology

Plant phenology, flowering (floral buds and flowers at anthesis) and fruiting (immature and mature fruits) was observed weekly. We used the activity index method for monthly assessment of phenophase presence or absence, indicating the percentage of individuals in the population, undergoing a phenological event [24], and the mean number of buds, flowers, green fruits, and mature fruits. Climatic data were obtained in the Meteorological Station of Ivinhema-state of Mato Grosso do Sul.

### 2.3. Floral Biology in the Field

We collected, from 20 adult *L. nervosa* plants, 60 flowers at different stages of development: pre-anthesis (n = 20), anthesis (n = 20) and post-anthesis or senescence (n = 20). The flower buds were measured in length from the pedicel to the terminal end. We recorded flower anthesis duration in hours, anther dehiscence, and floral longevity through direct observations. The presence of floral odor was verified using the method proposed by Dafni [25], which consists of leaving flowers in a capped vial and perception of the odors by the smell (human perception) after 24 h [26]. The presence and position of osmophores was detected using the method proposed by Oliveira-Filho [27], through the application of neutral red dye on floral parts, so that the stained parts correspond to the location of the osmophores. Stigma receptivity was assessed by applying hydrogen peroxide (3%) to stigmas and observing the presence of air bubbles, which indicates peroxidase activity [28]. We assumed stigmas to be viable and receptive to supporting pollen germ tube growth if peroxidase activity was apparent. The study of pollen stainability was carried out by optical microscopy, through which we analyzed anthers from different plants (n = 12) collected at different phases of development (pre-anthesis, anthesis, and post-anthesis). We used six histological slides and 400× magnification for each floral stage. Pollen stainability was determined by staining the cytoplasm with acetic carmine drops [29]. The stained grains were assumed viable.

### 2.4. Foraging Behavior of Floral Visitors

The insect samplings were carried out using a 30 cm diameter entomological net, for 11 weeks, in February, March, April, and May 2016. Floral visitors were recorded from our 72 focus plants while the collector (E.A.F.) performed active sampling by walking in a “Z” trajectory among plants.

Our records of bees were divided into six periods of 40 min each (6:00 a.m., 8:00 a.m., 10:00 a.m., 12:00 a.m., 2:00 p.m., and 4:00 p.m.). The remaining 20 min of each hour was dedicated to observing the behavior of floral visitors. Overall, 43.92 h were dedicated to the collection of bees and 21.96 h to the observation of the behavior of floral visitors. The bees were fixed in the field using ethyl acetate and observed under a stereoscopic microscope in the lab to certify the pollen they carried and later.

We documented floral visits (both the type of resource collected and the contact between the body of the visitor and the reproductive structures of the visited plant) through direct observation, photographic and video records. Bees were identified by a specialist. The bee vouchers that were collected can be found at the Museum of Biodiversity—MuBio, at the Federal University of Grande Dourados.

### 2.5. Reproductive Test and Pollen Limitation

We did a reproductive experiment to assess the Pollen Limitation Index (PLI). A total of 100 flowers in anthesis were randomly removed from 10 plants. The test started at 6:00 a.m., before the opening of the flowers. Fifty flowers of *L. nervosa* were isolated with bags of voile fabric white (0.5-mm mesh), and the other 50 were left unbagged. The 50 unbagged flowers were observed until fruit formation, so we were able to assess the production of fruits under natural conditions (open pollination), to monitor natural fruit formation and maturation. This was considered as the control treatment. The other 50 flowers were used to analyze fruit production from manual pollen transfer between different flowers of different plants (artificial-cross-pollination). In artificial-cross-pollination we bagged the flowers still in buds to avoid visiting pollinators and to wait for anthesis. After anthesis of flowers, pollen was collected from anthers of flowers with the help of a brush and was transferred to the stigma of different flowers from plants that had previously been bagged. It was established a minimum distance of 30 m between the donor (pollen) and recipient (stigma) plants. The procedure was performed only once. The flower was considered pollinated when the stigma was completely covered by pollen. Methodologies using voile tissue are often used in reproductive tests [30,31]. Fecundity, i.e., the ratio of the number of fruits to the number of flowers produced, was estimated at the final phase of fruit production in each treatment. Pollen limitation (the reduction in reproductive success due to an inadequate supply of pollen) [32] in *L. nervosa* was assessed by the pollen limitation index [PLI = 1 − (Fc/Fpc)]. Where *Fc* is the percentage of fruiting under natural conditions and *Fpc* is the percentage of fruiting by artificial-cross-pollination; where PLI values up to 0.2 indicate no pollen limitation, a PLI greater than 0.8 indicates extreme pollen limitation [33].

### 2.6. Evaluation of Seed

Ten fruits obtained by each pollination treatment were separated and the seeds were removed from them. All seeds from the same treatment were mixed and then separated into ten replicates of 75 seeds each (n = 750). Seeds were placed on a “germitest” filter paper, soaked in distilled water, and kept in a biochemical oxygen demand (BOD) germinator equipped with white-fluorescent lamps, constant temperature (25 °C) and with a photoperiod of 12 h. Seeds showing primary root protrusion were considered as germinated.

Germination was evaluated daily for 40 days. After this period, seedling root and shoot lengths were measured with digital calipers and the number of leaves recorded. We calculated the germination percentage (G), fallowing formula G = (n/N) × 100, where n = Total number of germinated seeds; N = Total number of seeds placed to germinate. The Mean Germination Time, was calculated in days, using the formula MGT= (∑*niti*)/∑*ni*, where *ni* is the number of seeds germinated in the interval between each count; and *ti* are the days counted from the start of the experiment [34]. We calculated Germination Speed Index (GSI) using the formula GSI = (G_1_/N_1_) + (G_2_/N_2_) + (G_3_/N_3_) + … + (G_n_/N_n_), where G1, G2, G3, …, Gn = number of seeds germinated in the first, second, third and last count; N1, N2, N3, …, Nn = number of days of sowing to the first, second, third and last count [35].

### 2.7. Statistical Analysis

The data were submitted to the shapiro to test for normality. With a Generalized Linear Mixed model, we tested if the number of visits (response variable) varied between honey bees and native wild visitors (pooled data) at different time points (fixed factors). In this model day of sampling was set as random variable and Poisson distribution, analysis was performed with the R package *lme4* v.1.0-6 [36]. The first observation at 6 am was excluded since no visitor was recorded at this time. The effect of treatment (pollination experiment-manuals vs. natural) was tested with Generalized Linear Model (GLM) with the frequency of fruit set as response variable. In this model we used a binary distribution of errors. Fruit length was compared between treatment with a linear model (LM). The number of seeds (response variable) was compared between treatment (fixed factor) in a GLM. Number of seeds that germinated were compared between treatment with LM. Yet, germination speed index (GSI) and mean germination time were compared between treatment with a GLM.

The variables root development (RT), number of leaves (NL) and shoot length (SL) are all significant correlated variables (Pearson, RT and NL, r = 0.14, t = 3.25, df = 533, *p* = 0.001; RT and SL, r = 0.27, t = 6.47, df = 533, *p* < 0.001; NL and SL r = 0.13, t = 3.25, df = 533, *p* = 0.001); thus, a GLM was developed only with shoot length as measure to determine the effect of treatment on size of newly germinated individuals. In this model the shoot length measures were set as response variable and pollination treatment (manual vs. natural) as fixed factor. 

The differences among the germination percentage of seeds, germination speed index and the mean germination time. The data were analyzed using the R language. We checked visually if model’s assumption were matched and in conformity with expectations with the package DHARma [37]. All analyses were performed in R [38]. with *p*-values lower than 0.05 (*p* < 0.05) considered as indicative of significant differences between the treatments. 

## 3. Results

### 3.1. Flowering and Fruiting Phenology

**Figure 2:**
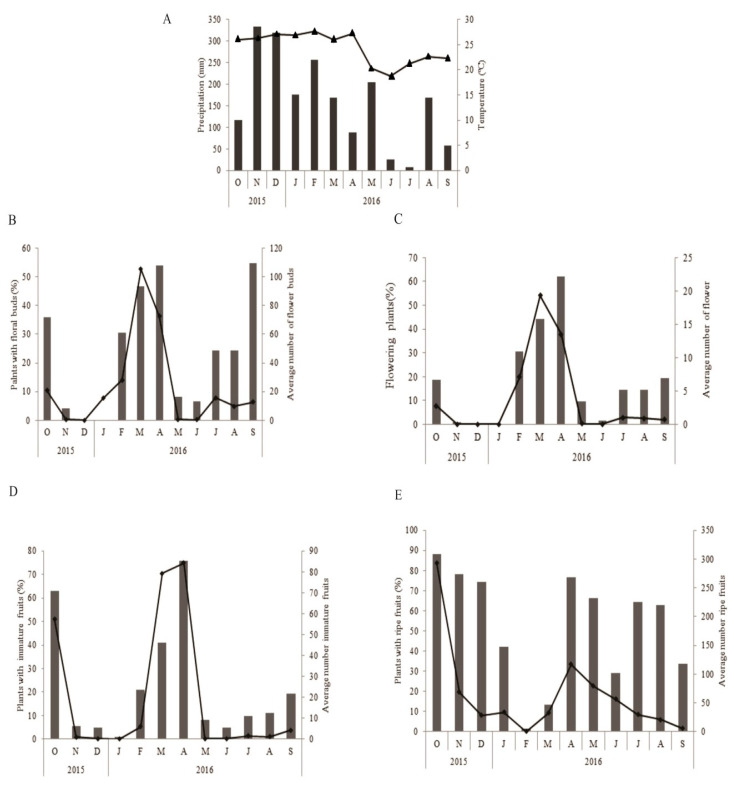
(**A**–**E**) Phenology of flowering and fruiting in *Ludwigia nervosa* from October 2015 to September 2016. (**A**) Climograph of Ivinhema, MS, during the period of phenological studies on a population of *Ludwigia nervosa*; Precipitation (mm) (light bar); Temperature (°C) (▲). (**B**–**E**) Percentage of plants in which a phenological event was occurring (dark bar); Mean number of phenophases (◆).

The period of maximum rainfall for the study region was between November 2015 and May 2016, and the highest average temperatures were recorded between November and April (Figure 2A). We recorded the greatest numbers of plants with buds at the end of the rainy season (April, 54.03%) and in the months preceding the rainy season (September, 54.83%) (Figure 2B). However, the average number of buds/plant was higher in March and April (Figure 2B). The percentage of plants with flowers was also highest in March (44.08%) and April (62.10%); in addition, the number of flowers per plant was also highest during these two months (Figure 2C). The highest number of plants with green fruit was recorded in October and April, with 62.9% and 75.1%, respectively, of individual plants bearing fruit (Figure 2D). The highest averages of green fruit/plant occurred in October, March, and April (Figure 2D). The plants carried ripe fruit almost the whole year, with peaks of production/plant in October (88.06%) and November (78.22%) (Figure 2E).

### 3.2. Floral Biology

**Figure 3:**
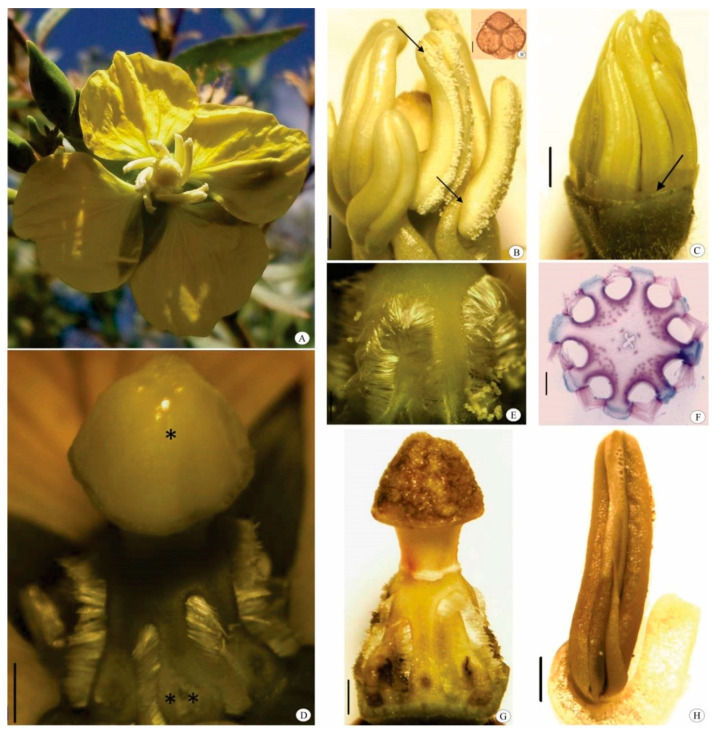
Some aspects of floral biology of *Ludwigia nervosa*. (**A**) Detail of floral morphology; (**B**) Dorsifixed anther (arrow) with longitudinal dehiscence (arrow) initiating pollen release, (**B’**) Pollen tetrad with viscin threads; (**C**) Corolla development (arrow); (**D**) Wet-looking stigma (*); Nectary (**); (**E**) Detail of nectary with trichomes; (**F**) Cross section of nectary; (**G**) Senescent gynoecium; (**H**) Senescent anther. Bar = 0.10 cm (**B**–**D**,**G**,**H**), 10 μm (**B’**), 0.05 cm (**E**), and 500 μm (**F**).

*Ludwigia nervosa* shows tetrameric, epigenetic, bisexual, actinomorphic, dichlamydeous flowers, which consist of a yellow dialypetalous corolla and a green gamosepalous calyx (Figure 3A). Each flower bears eight extrorse stamens, which are diplostemonous, heterodynamous, and dialystemonous. Anthers undergo longitudinal dehiscence and show a dorsifixed filament (Figure 3B). The ovary is tetralocular, tetracarpellate, and inferior. The placentation is axial and the fruit is polyspermic, dry, and dehiscent. Floral resources available are pollen tetrads and nectar.

In the bud phase (1.11 ± 0.07 cm in length), the corolla was being formed within the calyx, represented by a thin yellow membrane, and the light-colored stamens surrounding the stigma (Figure 3C). When buds reached 1.84 ± 0.14 cm, the thin corolla reached the apex of the buds, but was still completely covered by the calyx. When they reached 1.88 ± 0.09 cm, the petals of the flower buds could be seen between the sepals, but it was not possible to see either the stamens or the stigma from the exterior. At this stage, the stigma was covered by wet-looking papillae (Figure 3D), while the anthers were ready to initiate the release of whitish pollen grains.

In the pre-anthesis phase, the diameter of the corolla opening was 0.66 ± 0.20 cm, still much smaller than at anthesis, when the corolla diameter was 2.15 ± 0.62 cm (t = −7.9230; *p* < 0.0001) and the reproductive whorls were exposed. The stigma was capped, covered by papillae, wet and shiny. The floral nectaries had trichomes surrounding the base of the style between the stamens (Figure 3D–F).

Flowers anthesis began at 5 a.m. (fertile phase) and reached senescence at 1 p.m. (unfertile phase), at the time petals began falling off, followed by stamens. In post-anthesis, the stigma looked dark (Figure 3G), regardless of pollen presence, as did the anthers, which had little to no pollen left due to foraging (Figure 3H).

We found that *L. nervosa* flowers produced a strong, sweet odor. Osmophores were located in the anthers, in the outer region of the thecas, and in the filaments. Stigma receptivity began in pre-anthesis and extended until flower senescence. Pollen stainability was 82.87% in the pre-anthesis phase, 69.55% at anthesis, and 21.38% at post-anthesis. 

### 3.3. Foraging Behavior of Floral Visitors

**Figure 4:**
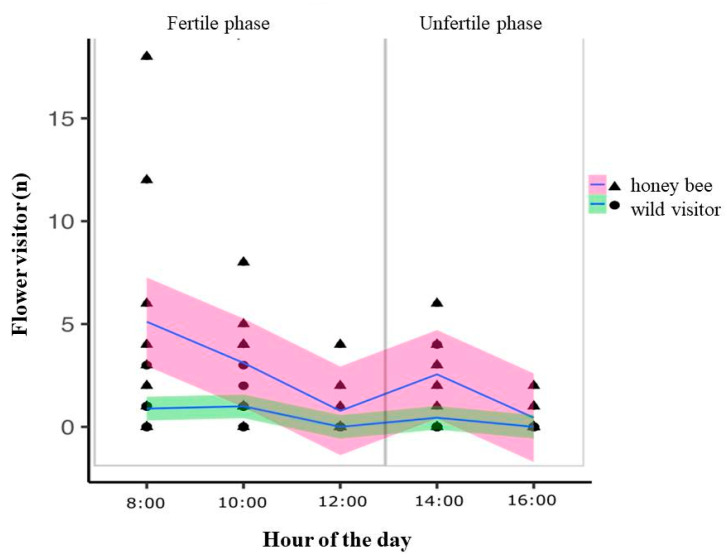
The number of honey bees visiting the flowers were significantly higher compared to all other wild native visitors together. The abundance of individuals varied significantly a long the day. Floral visits occurred mainly during the fertile phase of *L. nervosa*.

We collected 133 individuals of eight species of bees of the Apidae, Colletidae, and Megachilidae families. The bees collected were honey bees, *Apis mellifera* L., *Xylocopa* cf. *brasilianorum* L., *Xylocopa* cf. *frontalis* Olivier, *Exomalopsis analis* Spinola, *Exomalopsis fulvofasciata* Smith, F., *Melissoptila desiderata* Holmberg, *Tetraglossula bigamica* Strand, and *Megachile assumptionis* Schrottky. Most of the bees (98.5%) were considered potential pollinators. *Apis mellifera* was the predominant species visiting the flowers (85.82%) (Table 1). *A. mellifera*, *Xylocopa* ssp., and *M. (Schrottkyapis) assumptionis* bees carried out sternotribic pollination. The resources foraged by the bees were pollen and/or nectar (Table 1). Owing to their speed during the visits to the flowers, we could not record the resource collected by *M. desiderata* bees (Table 1). The number floral visitors on flowers of *L. nervosa* were significantly different between honey bees and all other native wild visitors (GLMM, χ^2^ = 48.23, df = 1, *p* < 0.001). The visitations differed significantly during the day (GLMM, χ^2^ = 50.15, df = 4, *p* <0.001) and was higher during the fertile phase of the L. nervosa (Figure 4).

Other non-bee species that occasionally visited flowers of *L. nervosa* were: *Bicyrtes* sp. (Crabronidae), *Polybia* sp. (Vespidae), *Campsomeris* sp. (Scoliidae), *Macraspis morio* Burmeister (Scarabaeidae), *Phymatinae* sp. (Reduviidae), and *Richardia* sp. (Platystomatidae). We recorded *M. morio* acting as a pillager visitor feeding on both the petal and reproductive whorls of *L. nervosa*.

### 3.4. Reproductive Test, Pollen limitation, and Evaluation of Seed

**Figure 5:**
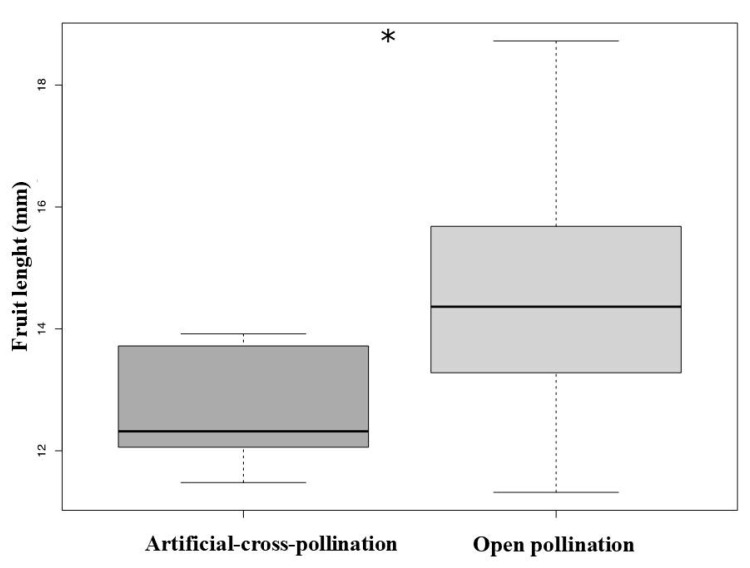
The fruit length produced from natural pollination experiment was significantly bigger compared to fruits of manual treatment (* *p* < 0.005).

Treatment (artificial-cross-pollination vs. open pollination) significantly affect the fruit set (GLM, χ^2^ = 26.17, df = 1, *p* < 0.001, Table 2) and fruit length (LM, F = 12.13, df = 1, *p* = 0.001, Figure 5, see Table 2). The number of seeds did not differ between treatments (GLM, χ^2^ *=* 1.85, df = 1, *p* = 0.17). There was no significant pollen limitation (PLI = −1.92).

Percent germination (G) was significantly higher in the seeds obtained by open pollination (LM, F = 5.68, df = 1, *p* = 0.02) (Table 3). However, there was no difference in GSI and MGT between treatments (GLM’s, χ^2^_GSI_ *=* 1.52, df = 1, *p* = 0.21; χ^2^_MGT_ *=* 0.31, df = 1, *p* = 0.57), see Table 3.

**Figure 6:**
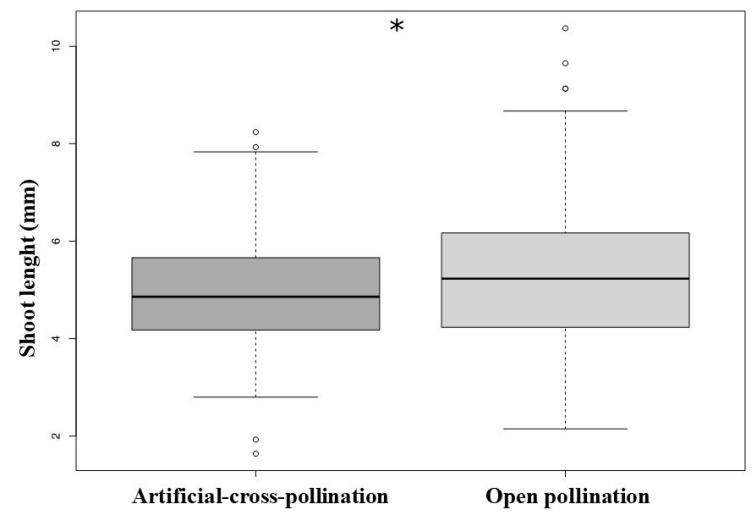
Growing rate, compared through the shoot length of newly germinated plants, was higher in the experiment of open pollination (natural pollination), (* *p* < 0.005).

There is a significant effect of treatment (manual vs. natural pollination) in the growing rate measured through shoot length (GLM, χ^2^ *=* 7.91, df = 1, *p* = 0.004, Figure 6). See average sizes for root sizes, shoot length, and number of leaves in the Table 4.

## 4. Discussion

In the current study we found that *L. nervosa* is attractive of several native bee species, however the exotic honey bee *Apis mellifera*, was the most common visitor. The high success of fertilization in the open (natural) pollination experiment and high frequency of honey bees, suggests that honey bees are the most important pollinator of this plant in the study site. Our data also shows that open pollination displayed an advantage compared to artificial (manual) pollination regarding fruit set, germination, and growing rate of newly germinated individuals.

### 4.1. Floral Biology

From the observation on the floral characteristics of *L. nervosa*, we categorized the species as pollination syndrome of the melittophily type, similarly to other *Ludwigia* species [2,3,13,14,39]. The flowers present diurnal anthesis, petals of yellow color, pleasant odor to the human perception, nutritional resources such as pollen grains arranged in tetrads [40] and are connected by viscin threads, which can vary in length, thickness, and structure [21]. The shape of the open flower suggests a landing platform for various bee species.

The wet aspect of the stigma observed in the early stage of flower development is due to the presence of papillary cells—with stigmatic secretion—which are also found in other species of the genus *Ludwigia* and are directly related to receptivity of stigma [41]. The stainability of *L. nervosa* pollen in the pre-anthesis, anthesis, and senescence phases of the flowers decreased throughout the day, probably due to the low relative humidity of the air and the high temperature, two factors that directly influence its viability [26].

In the *L. nervosa* flower, the position of the nectaries at the base of the gynoecium is advantageous for pollination because while accessing the nectar, flower visitors come into contact with the stigma and anthers, promoting the pollinating of the flower. The presence of osmophores also suggests the dependence of pollinator for reproduction [26]. Osmophores are commonly found in petals, sepals, or floral receptacles [42]. The floral traits of *L. nervosa* suggest that because of the fragile characteristics of the petals, the evolutionary process maximized the osmophores present in the filaments and teak of *L. nervosa* flowers, to attract pollinators for extended periods. Further studies are required to confirm this hypothesis.

### 4.2. Floral Visitor

Based on the literature, it was observed that in the different Brazilian regions the floral visitors to *Ludwigia* are diverse, which is probably due to the surrounding landscape and climate [2,3,13,22,39]. For instance, a total of 30 species was found in warm area and only 10 were recorded in colder environment [22] in São Paulo state, Brazil. Our observations were performed in a warm area, suggesting the reduced number of species (n = 8) may be a consequence of a highly anthropized areas. This seems to agree with the current literature regarding reduction of insect diversity at disturbed site [43], high frequency of honey bees and rarity of more specialized pollinators. Alternatively, the predominance of honey bees in our sample collection may also be due to its favorable localization of floral sources, densely populated nests [44], and eussocial behavior, with a very efficient communication system between the individuals of the colony [45]. Lastly, flowers with open morphology are frequently visited by generalist bees [46], which can further explain our findings. Interestingly we did not observed visits of stingless bees which are the native eusocial bees.

During the resource collection, *A. mellifera* had contact with the reproductive organs of the flowers, performing sternotribic pollination, i.e., pollen grains adhered to the ventral region of the pollinator’s body. Because pollen was collected from all anthers on the flowers, a great amount of pollen adhered to the ventral pilosity on the body of these bees. In this manner, pollen collected by visitors may increase the amount of pollen transferred to the stigma of the flowers they visit. The bees rested on the corolla, moved towards the nectary and inserted the mouthparts into the nectar chamber to collect nectar. The generalism of *A. mellifera* was also observed in *L. sericea* and *L. peruviana* [39], *L. elegans* [3,22,47] in the state of São Paulo and in the flowers of *L. tomentosa* in Guarapuava, Paraná [48]. Honey bees were considered an efficient pollinator due to its size, due to its frequent contact with the reproductive structures during nectar collection [22] and specially due its high visitation frequency.

All other bee species were non-eusocial and rare, but observation of the foraging behaviour suggest they might also work as pollinator. For instance, *Xylocopa* species are trapliners when visiting flowers, which optimizes cross-pollination [49]. In the present study, *X.* cf. *brasilianorum* and *X.* cf. *frontalis* touched the reproductive organs of *L. nervosa* for both pollen and nectar collection, owing to the large body size of the bees compared to the size of the flower. We did not identify the pollination ability of *Melissoptila desiderata* neither the floral resource it collected from flowers. Despite its small size, *Melissoptila* species are considered efficient pollinators other *Ludwigia* species [22,47]. Although, *Melissoptila bonaerensis* Holmberg, *M. paraguayensis* Brethes, and *M. setigera* Urban were sampled upon visiting *L. octavalvis* and *L. peruviana* flowers, however, no reports of bee their efficiency as pollinators were reported [4].

The *T. bigamica* bee is oligolectic (see [15]) for the genus *Ludwigia* [50]. In the present study, it was considered an effective pollinator despite its rarity. In all visits, this bee brought its scopa and pollen-covered tibia in contact with the reproductive organs of the flowers. The scopa of this species was adapted to handle pollen with viscin threads, allowing the collection and storage of the resource and facilitating its transfer to the stigma of the flower [47]. In *L. elegans*, this species was described as a specialized pollinator [47]. Bees collecting pollen from Onagraceae flowers are often specialized and oligolectic [22]. The bees *E. analis* and *E. fulvofasciata* were observed resting on the petals and drinking nectar. During their visits, they occasionally came into contact with the reproductive organs of the flowers as they attempted to collect nectar in more than one nectar chamber. However, our observations were limited to determine if this bee acted as a pollinator.

The *Megachile assumptions*, was considered an effective pollinator in the present study; it moved fast to collect resources and visited flowers of several plants while foraging. This behavior added to the ventral scope in megachilids seems to favor cross pollination in *Ludwigia* flowers. Similarly, *Megachile* aff. *brasiliensis* Dalla Torre and *M.* (Moureapis) *pleuralis* Vachal species were considered pollinators in *L. peruviana*, and *L. sericea*, respectively [39].

### 4.3. Reproductive Test, Pollen limitation, and Evaluation of Seed

Although self-incompatible plants are more vulnerable to the effects of fragmentation [7,8], the higher percentage of fruits produced under natural conditions and the absence of pollen limitation suggest that local pollinators contributed significantly to *L. nervosa* reproduction.

The absence of pollen limitation shows that there was sufficient and efficient deposition of pollen in *L. nervosa* flowers by floral visitors. Although the research site had been disturbed and was undergoing a regeneration process, our focus flowers were efficiently pollinated.

The effectiveness of cross pollination in *L. nervosa* was confirmed by the higher percent seed germination of seeds obtained through open pollination, which guaranteed the physiological quality of the seeds. Open pollination strongly influenced the development of seedlings (aerial part, root length, and number of leaves). Larger seeds may contain more reserves and offer competitive advantages for seedlings resulting from effective pollination [51,52].

The production of large numbers of viable seeds increases the chances of establishment after disruption [53]. Given the high percentage of germination, sexual reproduction in the area between a donor plant located far from the fertilized plant is an important mechanism for the propagation of the species and establishment of *Ludwigia* seedlings [17] and this reinforce the need of pollinators for *L. nervosa* reproduction. The effect of the distance separating the pollen donor plant from the recipient plant on the fruiting ratio seems to affect the reproduction of other pollinator-dependent species [54]. This mechanism may also contribute to genetic variability in the plant population, which is important for colonizing species that might be subject to environmental instability, as occurs in regenerated areas [55].

## 5. Conclusions

*Ludwigia nervosa* presents pollen and nectar as reward to floral visitors. Despite the disturbance in the study site, flowers were visited by several species. Successful plant reproduction suggests *A. mellifera* as its major effective pollinator due to its high abundance and displacement among flowers. Despite abundance of *L. nervosa*, native pollinators were rare highlighting the importance of conserved areas to sustain native bees.

## Figures and Tables

**Table 1:** Type of resource collected, behavior and percentage of floral visitors of *Ludwigia nervosa*. Captions: P—pollen, N—nectar, U—undefined, and PO—pollinator.

Floral Visitors	Resource Collected	Behavior	n	Individuals Collected (%)
HYMENOPTHERA				
APIDAE				
*Apis mellifera*	P/N	PO	115	85.82
*Xylocopa* cf. *brasilianorum*	P/N	PO	2	1.49
*Xylocopa* cf. *frontalis*	P/N	PO	2	1.49
*Exomalopsis analis*	P/N	U	7	5.22
*Exomalopsis fulvofasciata*	N	U	1	0.74
*Melissoptila desiderata*	U	U	2	1.49
COLLETIDAE				
*Tetraglossula bigamica*	P/N	PO	3	2.23
MEGACHILIDAE				
*Megachile assumptionis*	N	PO	2	1.49

**Table 2:** Fructification rate, average (±standard deviation), number of seeds and average (±standard deviation) size length of the fruits obtained from open pollination and artificial-cross-pollination treatments in *Ludwigia nervosa*.

Test	Flowers (n°)	Fruit Set (%)	Length of the Fruits (mm)	Number of Seeds
Artificial-cross-pollination	50	26.00 ± 0.44	12.83 ± 0.93	309.23 ± 116.1
Open pollination (control)	50	76.00 ± 0.43	14.53 ± 1.65	383.30 ± 183.3

**Table 3:** Percent germination (%G), germination speed index (GSI) and mean germination time (MGT) of *Ludwigia nervosa* seeds obtained from pollination treatments.

Test	% G	GSI	MGT
Artificial-cross-pollination	53.00 ± 31.06	1.23 ± 0.73	36.97 ± 8.09
Open pollination	78.00 ± 20.00	2.47 ± 2.06	37.26 ± 7.19

**Table 4:** Morphological data (average size ±standard deviation) of *Ludwigia nervosa* seedlings from seed germination test after specific pollination treatments. Measures in millimeters.

Test	Root Length	Shoot Length	Number of Leaves
Artificial-cross-pollination	1.85 ± 0.58	4.99 ± 1.22	2.00 ± 0.00
Open pollination	2.54 ± 1.17	5.31 ± 1.41	4.33 ± 1.00

## Data Availability

The data presented in this study are available on request from the corresponding author.
